# Commentary: Intestinal barrier function and immune homeostasis are missing links in obesity and type 2 diabetes development

**DOI:** 10.3389/fendo.2022.939703

**Published:** 2022-08-10

**Authors:** Yi-feng Wang, Song Wang, Hong-yang Xu, Li-jun Liu

**Affiliations:** ^1^ Department of Critical Care Medicine, The Affiliated Wuxi People’s Hospital of Nanjing Medical University, Wuxi, China; ^2^ Department of Emergency and Critical Care Medicine, Second Affiliated Hospital of Soochow University, Suzhou, China

**Keywords:** type 2 diabetes, hyperglycemia, intestinal barrier dysfunction, stress-induced hyperglycemia, crosstalk

We read with great interest the review by Riedel et al. highlighting the role of the intestinal system in the development of metabolic diseases (1). For a long time, type 2 diabetes (T2D) has been strongly bound to hyperglycemia. The comprehensive crosstalk summarized in the review is impressive and deeply advances our understanding of the interaction of T2D, obesity, metabolic inflammation, intestinal dysbiosis, and intestinal barrier dysfunction (IBD), leading us to reconsider the role of some pathophysiological changes beyond hyperglycemia in T2D. Therefore, IBD has caught our attention.

The intestinal barrier is a complex structure, while intestinal microbiota shape the biological barrier of the intestine. IBD causes increased intestinal permeability and allows undesirable luminal immunogens into the lymphatic and blood systems, finally contributing to systemic inflammation (2). In addition to gastrointestinal disease, the systemic inflammation caused by IBD drew our attention to metabolic diseases. Thaiss et al. investigated the IBD in mice with diabetes and obesity. Surprisingly, although IBD was associated with these two diseases, it was hyperglycemia, the most significant symptom of T2D, that independently drove IBD (3). Then our clinical study confirmed the relationship between IBD and hyperglycemia that the serum level of intestinal fatty acid-binding protein, a biomarker of IBD, was positively associated with the duration of hyperglycemia in T2D patients, with further investigation suggesting that the IBD worsened with the progression of T2D (4).

Besides T2D, hyperglycemia and IBD are also frequently observed in critical illnesses. Since the intestine is the motor of multiple organ dysfunction, the compromised intestinal barrier is involved in the development and deterioration of a critical illness (5). In addition to the irreversible hyperglycemia of T2D, reversible hyperglycemia, also known as stress-induced hyperglycemia (SIH), is common in critical illnesses. Interestingly, nondiabetic patients who suffered from SIH during a critical illness were more likely to get T2D in the future (6, 7), which is also observed in the most recent COVID-19 (8). In other words, with the IBD caused by hyperglycemia, reversible hyperglycemia (SIH) will result in irreversible hyperglycemia (T2D).

At first glance, it is not easy to accept the causal relationship between SIH and T2D. As is well known, there is a prediabetes stage between patients with and without T2D, which strongly indicates that hyperglycemia is a gradual process. Given the crosstalk of the intestinal system and metabolic diseases (1), SIH and T2D could be considered two different stages of hyperglycemia accompanied by systematic inflammation, IBD, and other pathophysiological changes. In other words, instead of having a causal relationship where one condition leads to the other, SIH and T2D might be hyperglycemia with a different severity.

Knowing that hyperglycemia is not unique to T2D, it is time to reconsider the role of hyperglycemia in T2D. When we talk about the management of a certain disease, both prevention and treatment should be included. Unlike the diverse recommendations on the prevention of T2D, the treatment of T2D is paradoxically limited to controlling hyperglycemia. In addition, only those agents with the capability of lowering blood glucose are qualified to be called antidiabetic drugs. With the crosstalk of the intestinal system and metabolic diseases mentioned in Riedel’s review (1), temporarily dealing with hyperglycemia is unable to solve all problems of T2D since other pathophysiological changes like obesity, metabolic inflammation, and IBD are still going to cause adverse consequences. Although blood glucose is easy to monitor, it is far from being sufficient for the treatment of T2D.

Too much attention has been paid to the management of hyperglycemia during T2D treatment, although hyperglycemia is only one part of the complex reciprocal causation of the intestinal system, metabolic diseases, and associated pathophysiological changes (1). A new perspective then comes to us that T2D is more likely to be a syndrome comprising hyperglycemia, insulin resistance, systematic inflammation, IBD, etc., rather than a single disease. Since hyperglycemia is a gradual process, it is more suitable to treat hyperglycemia as a concomitant symptom rather than the central one in T2D. Fortunately, in addition to controlling hyperglycemia, some antidiabetic drugs also have the effects of reducing insulin resistance, anti-inflammation, normalizing intestinal microbiota, and alleviating IBD (9), thus bringing additional benefits to T2D patients. Now, with the documented associations with hyperglycemia, inflammation, and other pathophysiological challenges, IBD might be a promising target during the management of T2D.

After all, this review inspires us to update our understanding of T2D. It is time to shift our focus from hyperglycemia alone to include the accompanying pathophysiological changes summarized in the crosstalk (1). By challenging the centrality of hyperglycemia and highlighting the significance of IBD, we propose innovation in the theory and management of T2D.

## Author contributions

YW and SW performed the review of literature, YW wrote the article, HX and LL conceived the commentary structure and revised the commentary, All authors contributed to the article and approved the submitted version.

## Conflict of interest

The authors declare that the research was conducted in the absence of any commercial or financial relationships that could be construed as a potential conflict of interest.

## Publisher’s note

All claims expressed in this article are solely those of the authors and do not necessarily represent those of their affiliated organizations, or those of the publisher, the editors and the reviewers. Any product that may be evaluated in this article, or claim that may be made by its manufacturer, is not guaranteed or endorsed by the publisher.
